# Cancer and Caregiving Among Older Black Americans and Families

**DOI:** 10.1093/geroni/igab046.182

**Published:** 2021-12-17

**Authors:** Katrina Ellis

**Affiliations:** University of Michigan, ANN ARBOR, Michigan, United States

## Abstract

Cancer disproportionately affects Black Americans and consequently, their families. In addition, cancer is often just one of the significant health concerns facing Black families at any one time. Research on family support after an adult cancer diagnosis overwhelmingly focuses on a single (i.e., primary) caregiver and spousal family caregivers, limiting understanding of the complexity of caregiving within family systems facing multiple health challenges. This presentation presents a framework for a broader focus on the role of family systems in providing cancer care, highlighting both the unique strengths and challenges facing Black families who provide care. A family comorbidity lens is used to underscore the need to better understand the nature and impact of concurrent health challenges within families. Ultimately, this perspective reflects the lived experiences of older Black adults and their families after a cancer diagnosis and has implications for future research and interventions to address health issues interdependently.

